# *Chlamydia pecorum* Associated With an Outbreak of Infectious Keratoconjunctivitis in Semi-domesticated Reindeer in Sweden

**DOI:** 10.3389/fvets.2019.00014

**Published:** 2019-02-05

**Authors:** Javier Sánchez Romano, Mikael Leijon, Åsa Hagström, Tomas Jinnerot, Ulrika K. Rockström, Morten Tryland

**Affiliations:** ^1^Arctic Infection Biology, Department of Arctic and Marine Biology, UiT Arctic University of Norway, Tromsø, Norway; ^2^Department of Microbiology, National Veterinary Institute, Uppsala, Sweden; ^3^Gård & Djurhälsan, Uppsala, Sweden

**Keywords:** alphaherpesvirus, *Chlamydia pecorum*, CvHV2, IKC, infectious keratoconjunctivitis, reindeer

## Abstract

Infectious keratoconjunctivitis (IKC), the most common ocular disease in ruminants worldwide, has affected semi-domesticated Eurasian reindeer (*Rangifer tarandus tarandus*) for over 100 years, both as individual cases and in outbreaks affecting tens to hundreds of animals. Recurrent IKC outbreaks have been affecting a semi-domesticated reindeer herd in Östra Kikkejaure (Norrbotten county, Sweden) from 2014. The latest episode of these recurrent outbreaks, in winter 2016/2017, was investigated in this study. Clinical findings were in line with previous reports of IKC in semi-domesticated reindeer and the clinical signs displayed by the affected animals (*n* = 30) included increased lacrimation, follicular conjunctivitis, purulent secretions around the affected eyes and corneal edema. Laboratory analyses of the samples revealed the presence of *Chlamydiaceae* in most samples obtained from the clinically affected animals (98.3%, *n* = 60), but also a high seroprevalence of cervid herpesvirus 2 (CvHV2) antibodies (56.6%, *n* = 53). *Moraxella bovoculi* was isolated from nine IKC-affected animals during the outbreak (45.0%, *n* = 20). All affected animals were treated with long-acting antibiotics and recovered from the disease, testing negative for the presence of *Chlamydiaceae* DNA by PCR 16 days and 3 months after the initial treatment. For the first time, *Chlamydia pecorum* was identified in semi-domesticated reindeer, and the involvement of *Chlamydiaceae* in a clinical outbreak of IKC is reported. The CvHV2 seroprevalence (56.6%) and the data obtained from a previous outbreak in 2014 also suggest the involvement of the reindeer alphaherpesvirus in the recurrent outbreaks.

## Introduction

Infectious keratoconjunctivitis (IKC) is regarded as one of the most common transmissible ocular diseases in ruminants worldwide, with numerous examples of domestic and wild susceptible species and a great variety of causative and presumably causative agents reported (1–10). IKC has been reported from the Fennoscandian herds of semi-domesticated Eurasian tundra reindeer (*Rangifer tarandus tarandus*) for more than 100 years (11), and nowadays is a rather common disease (12), appearing as individual cases or in outbreaks (13, 14). IKC in reindeer has been described as multifactorial and a variety of microorganisms have been identified in animals with clinical signs of the disease, including bacteria from the family *Chlamydiaceae, Moraxella bovoculi*, and *Mycoplasma conjunctivae*, and viruses, such as cervid herpesvirus 2 (CvHV2) and pestivirus (14, 15). CvHV2 and *M. bovoculi* were recently suggested as possible primary causative agents of the disease (14, 15) and the ability of CvHV2 as the primary causative agent of IKC in reindeer was demonstrated in an experimental inoculation experiment (16). However, other microorganisms are likely involved in the development of the disease in reindeer, but their role remains unclear.

Bacteria of the family *Chlamydiaceae* are widely distributed and highly prevalent in humans and animals, being the cause of ocular, reproductive, respiratory, and urinary tract diseases, and some of them representing zoonotic threats (17–19). The classification of this family was revised based on ribosomal RNA and divided it in two different genera, *Chlamydia* and *Chlamydophila* (20). However, a reversion of this taxonomic division has been recently proposed, with all species being included again in the genus *Chlamydia* (21, 22). Even though bacteria belonging to this family have not been described as causative agents of IKC in reindeer, several species of this obligate intracellular gram-negative bacteria have been linked to ocular disease in domestic sheep (*Ovis aries*) and goat (*Capra aegagrus hircus*) (4, 23–25), riverine buffalo (*Bubalus bubali*) (24), big horn sheep (*Ovis canadensis*) (8), red deer (*Cervus elaphus*) (7, 26), cat (*Felis silvestris*) (27, 28), koala (*Phascolarctos cinereus*) (29, 30), or guinea pig (31). Chlamydial keratoconjunctivitis in ruminants is, in the early stages, characterized by bilateral epiphora, chemosis, and conjunctival hyperemia, progressing to prominent conjunctival follicle formation and corneal neovascularization (32).

The aim of this study was to conduct a clinical investigation of semi-domesticated Eurasian tundra reindeer during an outbreak of IKC, to conduct relevant treatment of affected animals and to identify potential pathogens contributing to the development and spread of the disease.

## Materials and Methods

### Ethics Statement

No specific ethical permissions were required for this study. Sampling of the animals was conducted during clinical evaluation and for medical reasons with the objective of determining the cause of the clinical outbreak prior to treatment, with the approval of and in collaboration with the reindeer herders.

### Summary of the Outbreak (2014-2017)

**Figure 1:**
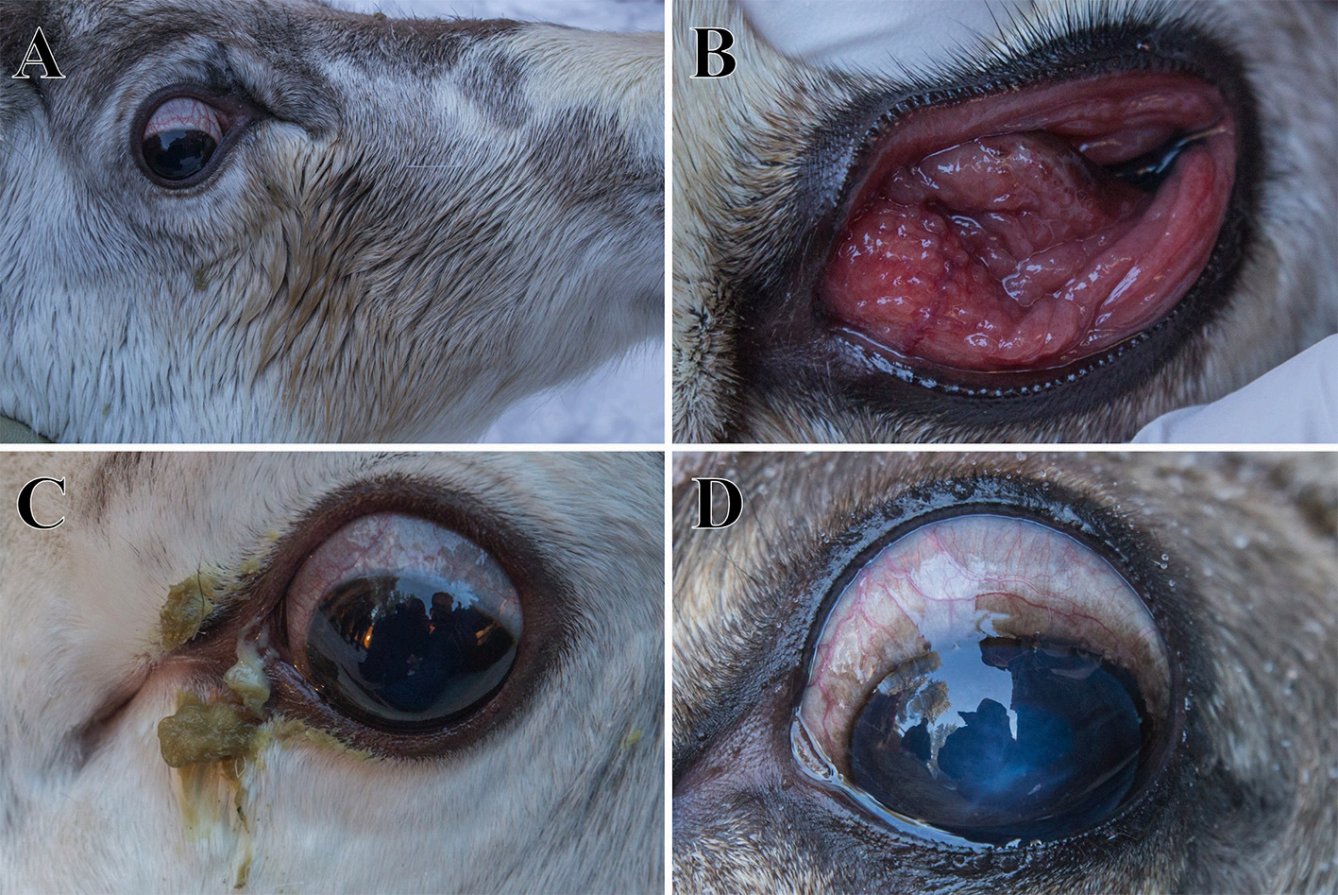
Clinical signs of infectious keratoconjunctivitis in semi-domesticated Eurasian tundra reindeer (*Rangifer t. tarandus*) observed during the clinical evaluation and sampling of animals in March 2017 in Östra Kikkejaure, Sweden. Animals displayed unilateral or bilateral increased lacrimation and ocular discharge shown as discoloration of the fur **(A)**, follicular conjunctivitis **(B)**, purulent secretions from the affected eyes **(C)**, and/or corneal edema, visible as an opaque cornea with a bluish coloration **(D)**.

The herders of a reindeer flock located at Östra Kikkejaure (Norrbotten county, Sweden) reported annual winter outbreaks of IKC during 2014-2017. The onset of outbreaks was associated with typical herding procedures, such as transport of animals between seasonal pastures, sorting, and selection of slaughter animals and marking of calves, during which the animals were herded into a fenced area for easier manipulation and control. For the outbreak during the winter 2016-2017, which will be described and addressed in detail in this study, the first few animals with clinical signs were spotted in November 2016, segregated from the main herd, which consisted of ~3000 reindeer, and examined. In February 2017, a few weeks after ~500 semi-domesticated reindeer were corralled for supplementary feeding with pelleted reindeer food, animals displaying early signs of infection were again spotted by the herders and segregated from the corralled flock. Upon clinical examination of affected animals, it was possible to identify the typical clinical signs of IKC, i.e., increased lacrimation (Figure 1A), follicular conjunctivitis (Figure 1B), purulent secretions around the affected eyes (Figure 1C) and corneal edema which was visible as a bluish, opaque coloration of the cornea (Figure 1D). Affected animals were sampled and treated, if needed.

### Sampling

During the first clinical examination, on November 16th 2016, animals displaying clinical signs of IKC were isolated from the rest of the herd in a fenced area. They were physically restrained and subjected to a clinical examination, including examination of the eyes with an ophthalmoscope and a fluorescein test, to identify corneal ulcers. A blood sample was obtained, and affected eyes were sampled by gently rubbing an eSwab (COPAN Italia S.p.A., Brescia, Italy) over the mucosal membrane of the conjunctival fornix.

The outbreak of IKC continued during the winter months and new veterinary investigations and sampling were carried out on March 1st and March 6th 2017, of 30 animals with IKC and one with no clinical symptoms (Table 1). The animals were again subjected to an ophthalmological investigation and sampled as described above. Animals diagnosed with IKC were treated with a subcutaneous injection of the long-acting antibiotic gamithromycin (150 mg/25 kg; Zactran®, Merial/Boehringer Ingelheim, Ingelheim am Rhein, Germany). The severity of IKC was classified as mild to moderate for animals with increased lacrimation, conjunctivitis and/or pus-like secretions, or severe if the animals displayed corneal opacity, corneal ulcer and/or severe panophthalmitis. Seventeen cases were defined as unilateral IKC and 13 cases as bilateral IKC. The affected animals ranged in age from calves of the year (~10-months-old) to ~4-year-old animals.

**Table 1:** Semi-domesticated reindeer investigated during the 2014 and 2016/2017 infectious keratoconjunctivitis outbreaks in Östra Kikkejaure.

**Date of sampling**	***n***	**Ocular clinical signs**			***Chlamydiaceae* PCR**	**Cervid herpesvirus 2**	
		**No**	**Yes**	**Unknown**		**PCR**	**Serology**
02.2014^a^	32	2	6	24	–	6/8 (75.0%)	23/24 (95.8%)
11.2016	28	–	–	28	–	–	20/28 (71.4%)
01.03.17	6	–	6	–	6/6 (100%)	0/6 (0%)	–
06.03.17	25	1	24	–	25/25 (100%)	4/25 (16.0%)	10/25 (40.0%)
22.03.17	7	7	–	–	0/7 (0%)	0/7 (0%)	–
27.06.17	9	9	–	–	0/9 (0%)	–	–

*Semi-domesticated reindeer investigated (n) by Taqman-based real-time PCR for the presence of Chlamydiaceae (n = 47) and cervid herpesvirus 2 (CvHV2) DNA (n = 46) and by enzyme-linked immunosorbent assay (Serology) (n = 77) for antibodies against alphaherpesvirus in Östra Kikkejaure during the IKC outbreaks of 2014 and 2016/2017. Data presented as Percentage (positive/tested)*.

a *Data obtained from Sánchez Romano et al. (15)*.

A follow up examination was performed on March 22nd. Seven of the animals investigated early in March were again sampled as previously described, and a second follow up examination was performed on June 27th, during which nine reindeer were examined and sampled.

Blood was sampled from the jugular vein with a vacutainer blood collection system (BD Vacutainer®; BD, Plymouth, UK), using tubes for full blood (serum) and tubes with K2 EDTA as anti-coagulant (plasma and white blood cells, buffy coat), and venoject needles (Terumo, Leuven, Belgium). Blood tubes were centrifuged at 3,500 rpm for 10 min and serum, plasma and buffy coat were collected and stored at −20°C.

For molecular investigations, swab samples were obtained by gently rubbing a swab (4N6 FLOQswabs® genetics; COPAN Italia S.p.A., Brescia, Italy) in the conjunctival fornix and placing the swab in a 1.8 ml cryotube with 800 μl of Eagle's minimum essential medium (EMEM), with antibiotics in final concentrations of 100 IU/ml of penicillin, 100 μg/ml of streptomycin, 50 μg/ml of gentamicin, and 2.5 μg/ml of amphotericin B, and stored at −80°C until further analysis.

For bacteriological investigations, a swab sample for collection and preservation of aerobic, anaerobic and fastidious bacteria (eSwab; COPAN Italia S.p.A., Brescia, Italy) was obtained from the conjunctival fornix, placed in its transport medium container and stored at 4°C until processed in the laboratory.

### Molecular Investigations

DNA was extracted from conjunctival swab samples (4N6 FLOQSwabs) with Maxwell 16® Buccal Swab LEV DNA purification kit (Promega, Madison, WI, USA), with slight modifications of the manufacturer's protocol. In summary, 300 μl of the swab storage media mixed with 30 μl Proteinase K and 300 μl Lysis buffer were incubated at 56°C for 20 min. Subsequently, 350 μl of the lysated mixture were added to the well #1 of the Maxwell® 16 LEV cartridge, which was then processed as indicated by the manufacturer.

#### *Chlamydiaceae* Real-Time PCR

DNA samples from one eye of 14 animals, and both eyes of 23 other animals (*n* = 60) displaying clinical signs of IKC were analyzed (National Veterinary Institute SVA, Sweden) by a TaqMan real-time PCR specific for members of the family *Chlamydiaceae*, targeting the 23S rRNA operon (33). The cut-off value, i.e. PCR cycle number above which a threshold cycle (C_t_) value is considered a false positive, was set at C_t_ > 38, with samples with a C_t_ below that being considered positive for the presence of *Chlamydiaceae* DNA. Eye swab samples from 17 asymptomatic reindeer were also analyzed (*n* = 21). As a positive control a stock of untyped *Chlamydiaceae* DNA derived from a clinical specimen was used according to the standard procedures at the molecular diagnostics division at SVA.

#### Cervid Herpesvirus 2 Real-Time PCR

A real-time Taqman Probe-based PCR, amplifying a 95 bp region of the *UL27* gene was performed as described previously (34) with slight modifications (16). Sixty-nine DNA samples extracted from eye swabs of 45 animals were run in duplicates, together with a positive control (CvHV2 DNA), non-template control (tissue from reindeer not exposed to CvHV2) and a negative control (water).

#### 16S rRNA Gene Library Preparation and NGS Amplicon Sequencing

Libraries of a ca 460 nt long amplicon carrying the variable V3 and V4 regions of the 16S ribosomal RNA gene were obtained according to the protocol 16S Metagenomic Sequencing Library Preparation (Rev.B) recommended and applied for the Illumina MiSeq System (Illumina Inc, San Diego). The amplicon libraries were combined at equimolar concentrations, spiked with 5% PhiX control (Illumina Inc, San Diego), denatured and loaded on a MiSeq flow cell and sequenced with a 600 cycles reagent kit v3 (Illumina Inc, San Diego) in paired-end sequencing runs.

#### 16s rRNA NGS Data Analysis

The MiSeq amplicon sequencing data were analyzed with the 16S amplicon data dedicated pipeline in the microbial genomics plugin module (version 2.5.1) of the CLC genomics workbench (version 11). The reads were trimmed based on a Phred quality score lower limit of 13 (i.e., 5% error probability) and adapter sequences were removed. The Paired end reads were merged and verified to be of identical length before operational taxonomical unit clustering using the SILVA OTU 16S database v. 123 with 97% as required similarity for assignment (35). Sequence data can be accessed with the accession number PRJNA512530.

#### *Chlamydia* Phylogeny

The maximum likelihood method was used for creating a phylogenetic tree using the UPMGA method for creating a starting tree and the HKY five-parameter nucleotide substitution model was used for estimating relationships using the CLC genomics workbench algorithm in the slow and very accurate modes. Discontinuous BLASTn were carried against the nt-database.

### Serology

Twenty-five serum samples obtained from the fenced reindeer (March 2017) and 28 serum samples previously collected from animals in the same herd were tested for the presence of antibodies against alphaherpesvirus with a commercial BoHV1 blocking enzyme-linked immunosorbent assay (bELISA) kit (LSI, Lissieu, France) previously validated for the testing of reindeer serum samples for CvHV2 specific antibodies (36). Positive and negative controls for cattle provided in the bELISA kit and for reindeer (36) were included on each plate.

### Bacteriological Investigations

eSwab samples obtained from the conjunctiva of 41 reindeer, 24 with and 17 without clinical signs of IKC, were remitted to the Swedish National Veterinary Institute (SVA) for routine general aerobic bacteriological diagnostics. Colonies of interest were subcultured for purity and typed with MALDI-TOF mass spectrometry.

## Results

### Molecular Investigations (Table 1)

All but one swab samples from IKC affected reindeer investigated for the presence of *Chlamydiaceae* DNA were positive (98.3%, *n* = 60). In contrast, it was only possible to identify *Chlamydiaceae* DNA in two eye swabs (*n* = 21) obtained from one of the 17 animals without clinical signs of the disease (9.5%, *n* = 21). Positive swabs from reindeer without clinical signs of IKC were obtained from an animal in direct contact with affected reindeer, while animals sampled later during the outbreak (3 weeks and 3.5 months) and were all negative for the presence of *Chlamydiaceae* DNA.

CvHV2-specific DNA (real-time PCR) was detected in the eye swabs of four reindeer with clinical signs of IKC (10.8%, *n* = 37) and in none of the animals without clinical signs of the disease (0%, *n* = 8).

#### 16S rRNA Gene and Nucleotide Sequencing

**Figure 2:**
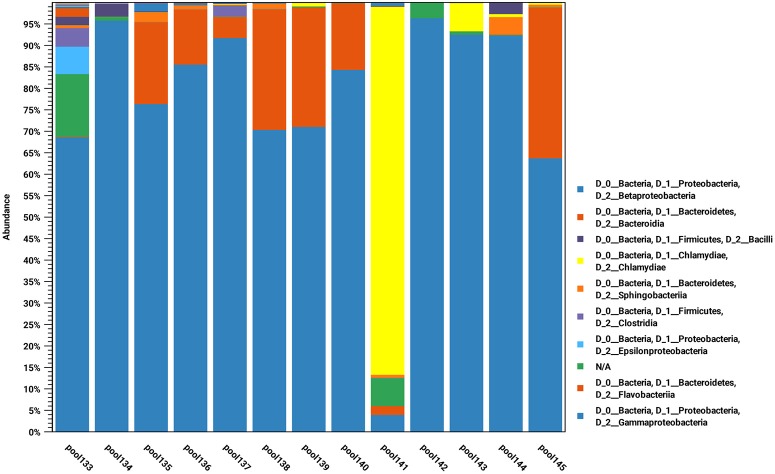
The bacterial populations derived from 16S rRNA sequence classification for the indicated eye swab pools. The populations are displayed aggregated and colored at the taxonomic class and phylum levels, respectively.

**Figure 3:**
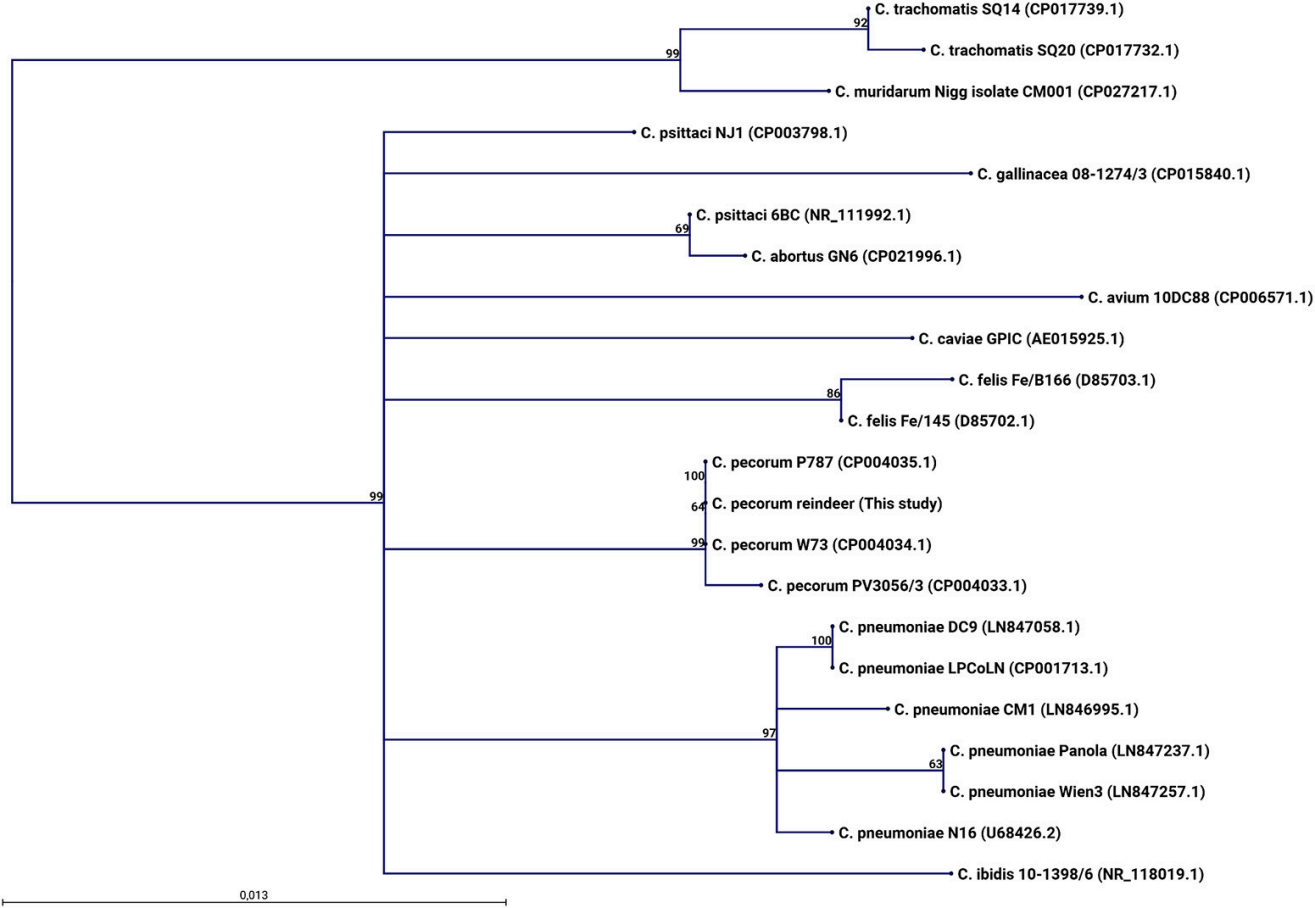
A maximum likelihood phylogenetic tree based on alignment of a 427 nucleotides segment of the 16S rRNA gene V3 and V4 variable regions was inferred using the HKY model of sequence evolution. Bootstrap support values > 50 percent obtained from 1,000 replications are indicated next to the relevant nodes. Sequences for the reference *Chlamydia* species obtained by discontinuous BLASTn searches against the NCBI GenBank database were aligned with the reindeer *Chlamydia* 16S rRNA gene sequence generated in this study, using the CLC genomics workbench algorithm in the slow and very accurate mode. The P787 (CP004035.1) and W73 (CP004034.1) *C. pecorum* strains, both isolated from sheep, are identical to the *C. pecorum* reindeer identified from six DNA pools of 12 animals with clinical signs of IKC in this study, across the gene regions characterized.

Samples from 10 animals from 2017 and four animals from 2014 where investigated by high-throughput amplicon sequencing, by pooling the DNA samples in pairs, including six pools in which samples from both eyes of a single animal were pooled together, and six pools in which one sample from two animals were pooled together. All pools except two were found to contain *Chlamydiaceae* DNA (Figure 2). Comparison with SILVA database v123 using the CLC genomics workbench revealed that the most similar sequence was *Chlamydia pecorum*. In addition, all samples contained an identical dominating *Chlamydia* sequence variant. When this sequence was analyzed using discontinuous BLASTn against the nt-database, the assignment to *Chlamydia pecorum* was confirmed (Figure 3).

### Serology (Table 1)

Overall CvHV2 seroprevalence during the 2016/2017 outbreak was 56.6% (*n* = 53). From animals in which the clinical signs were registered (*n* = 25), 41.7% of clinically affected animals (*n* = 24), and none of the animals that were not clinically affected (0%, *n* = 1) were seropositive. All adults (100%, *n* = 3) were seropositive, while for calves the seroprevalence was 31.8% (*n* = 22).

### Bacteriological Cultivation

Of the 41 reindeer sampled for bacteriology, it was possible to cultivate bacteria of different species from the conjunctiva of 32 animals, from which 20 displayed clinical signs of IKC, and 12 did not show any signs of the disease (Table 2). Beta-haemolytic *Moraxella bovoculi* colonies were isolated from seven of the 20 animals showing clinical signs of IKC (35.0%) and from one out of 12 with no such signs (8.3%) in pure culture. Furthermore, *M. bovoculi* was isolated from two additional animals with clinical signs of IKC in mixed culture with *Staphylococcus* sp. and *Klebsiella oxytoca* (2/20, 10.0%).

**Table 2:** Bacteria isolated from the eyes of semi-domesticated reindeer during the 2016-2017 infectious keratoconjunctivitis outbreak.

**Bacterial species**	**n**	**IKC +**	**IKC –**
*Moraxella bovoculi*	8	7/20 (35.0%)	1/12 (8.3%)
*M. bovoculi* + *Staphylococcus* sp.	1	1/20 (5.0%)	0/12 (0%)
*M. bovoculi* + *Klebsiella oxytoca*	1	1/20 (5.0%)	0/12 (0%)
*Staphylococcus* sp.	1	1/20 (5.0%)	0/12 (0%)
*Escherichia coli*	1	1/20 (5.0%)	0/12 (0%)
*Acinetobacter baumanii*	1	1/20 (5.0%)	0/12 (0%)
*Pseudomonas fulva*	3	0/20 (0%)	3/12 (25.0%)
*Staphylococcus aureus*	2	0/20 (0%)	2/12 (16.7%)
*Klebsiella oxytoca*	1	0/20 (0%)	1/12 (8.3%)
Mixture of bacteria (unidentified)	13	8/20 (40.0%)	5/12 (41.7%)
Total	32	20	12

*Number of swabs from the eyes of semi-domesticated Eurasian tundra reindeer (Rangifer tarandus tarandus) (n) with (IKC +) and without (IKC –) clinical signs of infectious keratoconjunctivitis from which bacteria were isolated. Data presented as Percentage (positive/tested)*.

## Discussion

Aside from the presence of follicular conjunctivitis, the clinical signs and the development of the outbreak of IKC in semi-domesticated reindeer reported here are concordant with previous descriptions (11, 13, 14, 37), and with IKC outbreaks in the same area reported in the 1970s (38).

However, follicular conjunctivitis was the most dominant and characteristic sign during the 2016/2017 outbreak. While follicular conjunctivitis has been described as one of the characteristic clinical signs in herpesvirus conjunctivitis caused by feline herpesvirus 1 in cats (39, 40), herpes simplex virus in humans (41, 42), and equine herpesvirus 2 in horses (43), it is also a characteristic feature of chlamydial conjunctivitis caused by *Chlamydia psittaci, Chlamydia pneumoniae, Chlamydia felis*, or *Chlamydia caviae* (31, 42, 44, 45).

The control of the outbreak, by treatment of the animals with macrolides, together with the ubiquitous detection of *Chlamydiaceae*-specific DNA in 59 out of 60 swab samples obtained from the eyes of affected animals (98.3%), suggested a member of this family were involved in the development of the clinical signs during this outbreak. This finding was also supported by the absence of DNA of these bacteria in the ocular swabs from animals after treatment with antibiotics and recovery from the disease (0%, *n* = 16). The 16S ribosomal RNA sequence characterization of at least six of the animals indicated the presence of *C. pecorum* as the dominant *Chlamydiaceae* species during the outbreak. To our knowledge, this is the first report of *C. pecorum* being involved in an IKC outbreak in semi-domesticated reindeer. Even though bacteria of this family were previously identified in reindeer with clinical signs of IKC, no association between the presence of these bacteria and the presence of clinical signs of IKC has previously been established (15).

CvHV2 seroprevalence (57.7%, *n* = 52) indicated that the virus is enzootic in reindeer in this region, as described for other reindeer and caribou populations (46–49).

It has been reported that the replication and copy numbers of CvHV2 during an active infection in the conjunctival mucosa of a reindeer may cease as the mucosal cell lining is destroyed and the severity of the secondary bacterial infections increase (14). This could also be the case in the 2016/2017 outbreak of IKC in Kikkejaure. During the first recorded IKC outbreak in this herd (2014) the seroprevalence of CvHV2 was 95.8% (Table 1) and CvHV2 DNA was detected in all ocular swabs obtained from IKC affected animals (100%, *n* = 6) (15). In contrast, during the IKC outbreak in the same herd that was investigated in November 2016 and March 2017, demonstrated a low prevalence of CvHV2 DNA in eye swabs (5.8%, *n* = 69), whereas the seroprevalence was 56.6% (*n* = 53). The high seroprevalence (CvHV2) and prevalence of CvHV2 DNA in ocular swabs in 2014 and the high seroprevalence during the 2016/2017 outbreak suggest that we cannot discard the virus as the initial trigger of the IKC outbreaks in this herd, possibly becoming less dominant and less important for the spread of IKC once the chlamydial infection took over. Further, the seroprevalence of CvHV2 in calves (31.8%, *n* = 22) was significantly higher than the 4–15% previously reported in semi-domesticated reindeer calves (49–52), suggesting that an active CvHV2 infection was circulating within the herd.

Previous serological screenings with seroprevalences of 21.6 and 30.8% suggested that chlamydial infections are present in semi-domesticated reindeer in Fennoscandia, but the low number of animals investigated (*n* = 291 and *n* = 26) and the lack of clinical signs associated to the infection made it difficult to determine if this pathogen is enzootic in reindeer in this region or if it is sporadically transmitted from other hosts (53, 54).

*Chlamydia abortus* and *C. psittaci* are the main zoonotic chlamydial species, causing severe symptomatology when infecting humans, i.e., septic infection and abortions, respiratory infections and pneumonia, myocarditis and encephalitis (18, 55). However, zoonotic chlamydial conjunctivitis with cat origin (*C. felis*) have been reported (44, 56), and little is known about the zoonotic potential of the other species of *Chlamydiaceae* (18). Because of this, the zoonotic potential of the reindeer strain of *C. pecorum* involved in this IKC outbreak should not be ignored, and preventive measures while working with affected animals should be taken into consideration. With this information in mind, further research will aim to genotype the strain identified in this study in order to evaluate its pathogenic potential.

The presence of *M. bovoculi* in the eyes of 10 animals was in line with previous reports (14, 15), and these findings strengthens the position of this bacterium as an opportunistic ocular pathogen in reindeer. Dickey et al. (57) described large genomic differences between isolates of *M. bovoculi* from cattle, suggesting that some strains may be commensal bacteria of the ruminant nasopharynx, while others may have an increased potential to cause keratoconjunctivitis. Until further characterization of the pathogenicity of the *M. bovoculi* isolates from reindeer, we cannot exclude the importance of *M. bovoculi* in the development of clinical cases of IKC in semi-domesticated reindeer.

From the eye swabs of 13 of the investigated animals (40.6%, *n* = 32), it was not possible to identify a dominant bacterial species, a result that matched previously reported bacteriological investigations in reindeer (14, 15). The mixture of bacterial species isolated in the eyes of reindeer during this outbreak, such as *Pseudomonas fulva, Staphylococcus* sp., and *Klebsiella oxytoca*, were most probably due to contamination of the eyes with bacteria from the skin or the environment, and do not seem to represent specific pathogens that are associated with the presence or absence of the disease.

Infectious keratoconjunctivitis outbreaks are often linked with the gathering and corralling of animals in high densities (13, 14, 58, 59). The outbreaks reported here are not an exception. In this case the gathering of animals at the end of autumn may have created the “perfect storm”: the stress of the animals leading to their immunosuppression that, together with the high density of animals and a population of naïve individuals, may contribute to the transmission of pathogens and establishment of the disease.

Due to the multifactorial nature of the disease, there is no specific treatment for IKC in reindeer, and while the recommendation is to recognize the early signs of the disease, isolate the affected animals and seek veterinary help, most herders slaughter the animals as soon as the disease is detected so that the meat can be used for consumption (12). During the outbreak 2016/2017, the early detection of *Chlamydiaceae* after the veterinary diagnosis in February, helped to choose the appropriate antimicrobial treatment. Tetracyclines, macrolides and quinolones are the antibiotics of choice for treatment of chlamydial infections in human and veterinary medicine (60). The special characteristics of reindeer herding and the challenge of conducting repeated medical treatment contributed to choosing a long-acting macrolide. All treated animals recovered from the disease and it was not possible to detect DNA specific for *Chlamydiaceae* in nine animals that were treated with antibiotics, recovered and were sampled 3 months after the initial treatment (0%, *n* = 12), which suggests a complete elimination of the chlamydial infection and not only a temporary reduction of the chlamydial load as it may happen when short-term antimicrobial therapies are used (61, 62).

## Conclusions

The clinical signs reported during this outbreak of IKC in reindeer were indistinguishable from previous reports of IKC in reindeer, which underlines the difficulties of diagnosing the causative agent of an outbreak solely based on clinical observations.

To our knowledge, this is the first report of *Chlamydiaceae* being involved in a clinical outbreak of IKC in reindeer. This is also the first time *C. pecorum* has been reported in semi-domesticated reindeer. Even though *Chlamydiaceae* DNA was detected in all the animals with clinical signs of IKC, further investigations are necessary in order to clarify the role of these bacteria in the development of IKC in reindeer, and whether they may act as a transmissible and causative agent of IKC.

The chlamydial species identified during the outbreak, *C. pecorum*, may be transmitted to other species, and reindeer herders, veterinarians, and other people working with infected semi-domesticated reindeer should comply with preventive measures while working with affected animals to avoid exposure to this pathogen and the infection of other susceptible species in the area, such as other cervid species or sheep. If properly diagnosed, chlamydial keratoconjunctivitis in semi-domesticated reindeer can be treated with macrolides, leading to a complete recovery of infected animals in early stages of the disease.

## Author Contributions

JS conducted the CvHV2 analyses, organized the dataset, and wrote the first draft of the manuscript. MT and UR secured funding for the study and organized the sampling. MT, UR, and JS contributed to the sampling of animals. ML, ÅH, and TJ conducted the bacteriology and *Chlamydiaceae* analyses. All authors contributed to the writing and accepted the final version of the manuscript.

### Conflict of Interest Statement

The authors declare that the research was conducted in the absence of any commercial or financial relationships that could be construed as a potential conflict of interest.

## References

[1] Åkerstedt J Hofshagen M. Bacteriological investigation of infectious keratoconjunctivitis in Norwegian sheep. Acta Vet Scand. (2004) 45:19–26. 10.1186/1751-0147-45-19 15535083 PMC1821000

[2] Belloy L Janovsky M Vilei EM Pilo P Giacometti M Frey J. Molecular epidemiology of *Mycoplasma conjunctivae* in Caprinae: transmission across species in natural outbreaks. Appl Environ Microbiol. (2003) 69:1913–9. 10.1128/AEM.69.4.1913-1919.2003 12676664 PMC154764

[3] Egwu GO Faull WB Bradbury JM Clarkson MJ. Ovine infectious keratoconjunctivitis: a microbiological study of clinically unaffected and affected sheep's eyes with special reference to *Mycoplasma conjunctivae*. Vet Rec (1989) 125:253–6. 10.1136/vr.125.10.253 2678719

[4] Gupta S Chahota R Bhardwaj B Malik P Verma S Sharma M. Identification of *Chlamydiae* and *Mycoplasma* species in ruminants with ocular infections. Lett Appl Microbiol. (2015) 60:135–9. 10.1111/lam.12362 25421836

[5] Marco I Mentaberre G Ballesteros C Bischof DF Lavín S Vilei EM. First report of *Mycoplasma conjunctivae* from wild *Caprinae* with infectious keratoconjunctivitis in the Pyrenees (NE Spain). J Wildl Dis. (2009) 45:238–41. 10.7589/0090-3558-45.1.238 19204357

[6] Mavrot F Vilei EM Marreros N Signer C Frey J Ryser-Degiorgis MP. Occurrence, quantification, and genotyping of *Mycoplasma conjunctivae* in wild Caprinae with and without infectious keratoconjunctivitis. J Wildl Dis. (2012) 48:619–31. 10.7589/0090-3558-48.3.619 22740528

[7] Gortázar Zaragoza C Fernández-de-Luco D Frölich K Keratoconjunctivitis in a free-ranging red deer (*Cervus elaphus*) population in Spain. Z Jagdwissensch. (1998) 44:257–61. 10.1007/BF02242031

[8] Meagher M Quinn WJ Stackhouse L. Chlamydial-caused infectious keratoconjunctivitis in Bighorn sheep of Yellowstone National Park. J Wildl Dis. (1992) 28:171–6. 1602566 10.7589/0090-3558-28.2.171

[9] Angelos JA *Moraxella bovoculi* and infectious bovine keratoconjunctivitis: cause or coincidence? Vet Clin North Am. Food Anim Pract. (2010) 26:73–8. 10.1016/j.cvfa.2009.10.002 20117543

[10] Galvão KN Angelos JA. Ulcerative blepharitis and conjunctivitis in adult dairy cows and association with *Moraxella bovoculi*. Can Vet J. (2010) 51:400–2. 20592830 PMC2839830

[11] Bergman A Contagious keratitis in reindeer. Skandinavisk Veterinärtidskrift (1912) 2:145–77.

[12] Tryland M Stubsjøen SM Ågren E Johansen B Kielland C. Herding conditions related to infectious keratoconjunctivitis in semi-domesticated reindeer: a questionnaire-based survey among reindeer herders. Acta Vet Scand. (2016) 58:22. 10.1186/s13028-016-0203-x 27068819 PMC4828894

[13] Rehbinder C Nilsson A An outbreak of kerato-conjunctivitis among corralled, supplementary fed, semidomestic reindeer [*Rangifer tarandus*] calves. Rangifer (1995) 15:9–14. 10.7557/2.15.1.1151

[14] Tryland M das Neves CG Sunde M Mørk T. Cervid herpesvirus 2, the primary agent in an outbreak of infectious keratoconjunctivitis in semidomesticated reindeer. J Clin Microbiol. (2009) 47:3707–13. 10.1128/JCM.01198-09 19726598 PMC2772613

[15] Sánchez Romano J Mørk T Laaksonen S Ågren E Nymo IH Sunde M Infectious keratoconjunctivitis in semi-domesticated Eurasian tundra reindeer (*Rangifer tarandus tarandus*): microbiological study of clinically affected and unaffected animals with special reference to cervid herpesvirus 2. BMC Vet Res. (2018) 14:15. 10.1186/s12917-018-1338-y PMC5771138 29338721

[16] Tryland M Sánchez Romano J Marcin N Nymo IH Josefsen TD Sørensen KK Cervid herpesvirus 2 and not *Moraxella bovoculi* caused keratoconjunctivitis in experimentally inoculated semi-domesticated Eurasian tundra reindeer. Acta Vet Scand. (2017) 59:23. 10.1186/s13028-017-0291-2 PMC5404682 28438213

[17] Burnard D Polkinghorne A. Chlamydial infections in wildlife - Conservation threats and/or reservoirs of ‘spill-over' infections? Vet Microbiol. (2016) 196:78–84. 10.1016/j.vetmic.2016.10.018 27939160

[18] Longbottom D Coulter LJ. Animal chlamydioses and zoonotic implications. J Comp Pathol. (2003) 128:217–44. 10.1053/jcpa.2002.0629 12834606

[19] Manavi K A review on infection with *Chlamydia trachomatis*. Best Pract Res Clin Obstetr Gynaecol. (2006) 20:941–51. 10.1016/j.bpobgyn.2006.06.003 16934531

[20] Everett KD Bush RM Andersen AA. Emended description of the order *Chlamydiales*, proposal of *Parachlamydiaceae* fam. nov. and *Simkaniaceae* fam. nov., each containing one monotypic genus, revised taxonomy of the family *Chlamydiaceae*, including a new genus and five new species, and standards for the identification of organisms. Int J Syst Bacteriol. (1999) 49:415–40. 10.1099/00207713-49-2-415 10319462

[21] Sachse K Bavoil PM Kaltenboeck B Stephens RS Kuo CC Rosselló-Móra R. Emendation of the family *Chlamydiaceae*: proposal of a single genus, *Chlamydia*, to include all currently recognized species. Syst Appl Microbiol. (2015) 38:99–103. 10.1016/j.syapm.2014.12.004 25618261

[22] Stephens RS Myers G Eppinger M Bavoil PM. Divergence without difference: phylogenetics and taxonomy of *Chlamydia* resolved. FEMS Immunol Med Microbiol. (2009) 55:115–9. 19281563 10.1111/j.1574-695X.2008.00516.x

[23] Constable PD Gay CC Hinchcliff KW Radostits OM Veterinary Medicine: A Textjournal of the Diseases of Cattle, Horses, Sheep, Pigs and Goats. 10th ed. Edinburgh: Saunders (2007).

[24] Osman KM Ali HA ElJakee JA Galal HM. Prevalence of *Chlamydophila psittaci* infections in the eyes of cattle, buffaloes, sheep and goats in contact with a human population. Transbound Emerg Dis. (2013) 60:245–51. 10.1111/j.1865-1682.2012.01337.x 22584046

[25] Polkinghorne A Borel N Becker A Lu ZH Zimmermann DR Brugnera E. Molecular evidence for chlamydial infections in the eyes of sheep. Vet Microbiol. (2009) 135:142–6. 10.1016/j.vetmic.2008.09.034 18945556

[26] Jelocnik M Self R Timms P Borel N Polkinghorne A Novel sequence types of *Chlamydia pecorum* infect free-ranging Alpine ibex (*Capra ibex*) and red deer (*Cervus elaphus*) in Switzerland. J Wildl Dis. (2015) 51:479–83. 10.7589/2014-08-220 25647593

[27] von Bomhard W Polkinghorne A Huat Lu Z Vaughan L Vögtlin A Zimmermann DR Detection of novel *Chlamydiae* in cats with ocular disease. Am J Vet Res. (2003) 64:1421–8. 10.2460/ajvr.2003.64.1421 14620780

[28] Sykes JE Studdert VP Anderson G Browning GF. Comparison of *Chlamydia psittaci* from cats with upper respiratory tract disease by polymerase chain reaction analysis of the ompA gene. Vet Rec. (1997) 140:310–3. 10.1136/vr.140.12.310 9106964

[29] Cockram FA Jackson ARB. Isolation of a *Chlamydia* from cases of keratoconjunctivitis in koalas. Austral Vet J. (1974) 50:82–3. 10.1111/j.1751-0813.1974.tb05265.x 4826002

[30] Girjes AA Hugall A Graham DM McCaul TF Lavin MF. Comparison of type I and type II *Chlamydia psittaci* strains infecting koalas (*Phascolarctos cinereus*). Vet Microbiol. (1993) 37:65–83. 10.1016/0378-113590183-8 8296453

[31] Lutz-Wohlgroth L Becker A Brugnera E Huat ZL Zimmermann D Grimm F. Chlamydiales in Guinea-pigs and their zoonotic potential. J Vet Med Ser A (2006) 53:185–93. 10.1111/j.1439-0442.2006.00819.x 16629952

[32] Smith BP Large Animal Internal Medicine. St. Louis, MI: Elsevier Health Sciences (2014).

[33] Everett KD Hornung LJ Andersen AA. Rapid detection of the *Chlamydiaceae* and other families in the order *Chlamydiales*: Three PCR tests. J Clin Microbiol. (1999) 37:575–80. 9986815 10.1128/jcm.37.3.575-580.1999 PMC84475

[34] Wang J O'Keefe J Orr D Loth L Banks M Wakeley P. Validation of a real-time PCR assay for the detection of bovine herpesvirus 1 in bovine semen. J Virol Methods (2007) 144:103–8. 10.1016/j.jviromet.2007.04.002 17561275

[35] Quast C Pruesse E Yilmaz P Gerken J Schweer T Yarza P. The SILVA ribosomal RNA gene database project: improved data processing and web-based tools. Nucleic Acids Res. (2013) 41:D590–D6. 10.1093/nar/gks1219 23193283 PMC3531112

[36] das Neves CG Roger M Yoccoz NG Rimstad E Tryland M Evaluation of three commercial bovine ELISA kits for detection of antibodies against Alphaherpesviruses in reindeer (*Rangifer tarandus tarandus*). Acta Vet Scand. (2009) 51:9. 10.1186/1751-0147-51-9 PMC2663558 19272136

[37] Oksanen A editor. Keratoconjunctivitis in corralled reindeer. In: Seventh Nordic Workshop on Reindeer Research. Tromsø (1993).

[38] Rehbinder C Clinical and epizootiological studies on keratitis in reindeer. Acta Vet Scand. (1977) 66:1–27. 271461

[39] Sykes JE Anderson GA Studdert VP Browning GF. Prevalence of feline *Chlamydia psittaci* and feline herpesvirus 1 in cats with upper respiratory tract disease. J Vet Intern Med. (1999) 13:153–62. 10.1111/j.1939-1676.1999.tb02172.x 10357102

[40] Rampazzo A Appino S Pregel P Tarducci A Zini E Biolatti B. Prevalence of *Chlamydophila felis* and feline herpesvirus 1 in cats with conjunctivitis in Northern Italy. J Vet Intern Med. (2003) 17:799–807. 10.1111/j.1939-1676.2003.tb02517.x 14658715

[41] Darougar S Hunter PA Viswalingam M Gibson JA Jones BR. Acute follicular conjunctivitis and keratoconjunctivitis due to herpes simplex virus in London. Br J Ophthalmol. (1978) 62:843–9. 10.1136/bjo.62.12.843 737165 PMC1043370

[42] Uchio E Takeuchi S Itoh N Matsuura N Ohno S Aoki K. Clinical and epidemiological features of acute follicular conjunctivitis with special reference to that caused by herpes simplex virus type 1. Br J Ophthalmol. (2000) 84:968–72. 10.1136/bjo.84.9.968 10966946 PMC1723617

[43] Miller TR Gaskin JM Whitley RD Wittcoff ML Herpetic keratitis in a horse. Equine Vet J. (1990) 22:15–7. 10.1111/j.2042-3306.1990.tb04703.x 9079109

[44] Hartley JC Stevenson S Robinson AJ Littlewood JD Carder C Cartledge J. Conjunctivitis due to *Chlamydophila felis* (*Chlamydia psittaci* feline pneumonitis agent) acquired from a cat: case report with molecular characterization of isolates from the patient and cat. J Infect. (2001) 43:7–11. 10.1053/jinf.2001.0845 11597148

[45] Lietman T Brooks D Moncada J Schachter J Dawson C Dean D Chronic follicular conjunctivitis associated with *Chlamydia psittaci* or *Chlamydia pneumoniae*. Clin Infect Dis. (1998) 26:1335–40. 10.1086/516373 9636859

[46] Tryland M Are we facing new health challenges and diseases in reindeer in Fennoscandia? Rangifer (2013) 32:35–47. 10.7557/2.32.1.2279

[47] das Neves CG Roth S Rimstad E Thiry E Tryland M Cervid herpesvirus 2 infection in reindeer: a review. Vet Microbiol. (2010) 143:70–80. 10.1016/j.vetmic.2010.02.015 20207086

[48] Evans AL das Neves CG Finstad GL Beckmen KB Skjerve E Nymo IH. Evidence of alphaherpesvirus infections in Alaskan caribou and reindeer. BMC Vet Res. (2012) 8:5. 10.1186/1746-6148-8-5 22243919 PMC3274481

[49] Kautto AH Alenius S Mossing T Becher P Belák S Larska M. Pestivirus and alphaherpesvirus infections in Swedish reindeer (*Rangifer tarandus tarandus L*). Vet Microbiol. (2012) 156:64–71. 10.1016/j.vetmic.2011.10.018 22078277

[50] das Neves CG Thiry J Skjerve E Yoccoz NG Rimstad E Thiry E Alphaherpesvirus infections in semidomesticated reindeer: a cross-sectional serological study. Vet Microbiol. (2009) 139:262–9. 10.1016/j.vetmic.2009.06.013 19604658

[51] Ek-Kommonen C Veijalainen P Rantala M Neuvonen E. Neutralizing antibodies to bovine herpesvirus 1 in reindeer. Acta Vet Scand. (1982) 23:565. 6301239 10.1186/BF03546775 PMC8295786

[52] Stuen S Krogsrud J Hyllseth B Tyler NJC Serosurvey of three virus infections in reindeer in northern Norway and Svalbard. Rangifer (1993) 13:215–9.

[53] Rehbinder C Nordkvist M Moreno JW A suspected virus infection of the oral mucosa in Swedish reindeer (*Rangifer tarandus* L). Rangifer (1985) 5:22–31.

[54] Neuvonen E. Occurrence of antibodies to group specific *Chlamydia* antigen in cattle and reindeer sera in Finnish Lapland. Acta Vet Scand. (1976) 17:363–9. 983896 10.1186/BF03547917 PMC8383949

[55] Rohde G Straube E Essig A Reinhold P Sachse K. Chlamydial Zoonoses. Deutsches Ärzteblatt Int. (2010) 107:174–80. 10.3238/arztebl.2010.0174 20358033 PMC2847324

[56] Ostler H Schachter J Dawson CR Acute follicular conjunctivitis of epizootic origin: feline pneumonitis. Arch Ophthalmol. (1969) 82:587–91. 10.1001/archopht.1969.00990020587003 5388669

[57] Dickey AM Loy JD Bono JL Smith TPL Apley MD Lubbers BV. Large genomic differences between *Moraxella bovoculi* isolates acquired from the eyes of cattle with infectious bovine keratoconjunctivitis versus the deep nasopharynx of asymptomatic cattle. Vet Res. (2016) 47:31. 10.1186/s13567-016-0316-2 26872821 PMC4752781

[58] Aschfalk A Josefsen TD Steingass H Müller W Goethe R. Crowding and winter emergency feeding as predisposing factors for kerato-conjunctivitis in semi-domesticated reindeer in Norway. Deutsche Tierärztliche Wochenschrift (2003) 110:295–8. 12910868

[59] Oksanen A Laaksonen S Hirvelä-Koski V Silmätulehdus nietosten keskellä porotarhassa. Suomen Eläinlääkärilehti (1996) 102:138–41.

[60] Walker E Lee EJ Timms P Polkinghorne A. *Chlamydia pecorum* infections in sheep and cattle: a common and under-recognised infectious disease with significant impact on animal health. Vet J. (2015) 206:252–60. 10.1016/j.tvjl.2015.09.022 26586214

[61] Andrews AH Goddard PC Wilsmore AJ Dagnell GJ. A chlamydial keratoconjunctivitis in a British sheep flock. Vet Rec. (1987) 120:238–9. 10.1136/vr.120.10.238 3576928

[62] Reinhold P Liebler-Tenorio E Sattler S Sachse K Recurrence of *Chlamydia suis* infection in pigs after short-term antimicrobial treatment. Vet J. (2011) 187:405–7. 10.1016/j.tvjl.2010.01.008 20800518

